# OA23 A complex case of psoriatic juvenile idiopathic arthritis

**DOI:** 10.1093/rap/rkae117.023

**Published:** 2024-11-05

**Authors:** Madeleine Mackay, Nanita Pai, Samundeeswari Deepak, Kapil Gargh, Satyapal Rangaraj, Kishore Warrier

**Affiliations:** Queens medical centre, Nottingham, United Kingdom; Queens medical centre, Nottingham, United Kingdom; Queens medical centre, Nottingham, United Kingdom; Queens medical centre, Nottingham, United Kingdom; Queens medical centre, Nottingham, United Kingdom; Queens medical centre, Nottingham, United Kingdom

## Abstract

**Introduction:**

This patient demonstrates many of the challenges that can be faced when treating juvenile idiopathic arthritis (JIA). The combination of difficult to treat disease, together with her social situation has made her case a particularly challenging one. Her case outlines the necessity of a multidisciplinary, and multi-agency, approach to treatment to ensure patient’s needs are met.

**Case description:**

This patient presented at five years old with a several year history of swelling in her knees and feet. On examination she had a polyarthritis affecting bilateral metacarpal-phalangeal joints, both wrists, both elbows, and both knees. She is ANA negative and RF negative.

She was initially treated with intravenous methylprednisolone (IVMP), methotrexate and joint injections. This patient’s disease has been very difficult to treat, with inadequate response to multiple medications (see table 1). She has had multiple flares, required 12 General anaesthetics for joint injections, as well as several pulses of IVMP to help control her inflammation.

The patient has persistent effusion of her knees, not responsive to intra-articular steroids and the presence of significant crepitus. An MRI of her right knee showed 14 mm loose bony fragment, in addition to synovitis and effusion. The orthopaedic team were not keen to manage this surgically.

She was discussed with other centres, following which combination of biologics / biologic with small molecule (JAK inhibitors) was felt to be the next option. Adalimumab was added to Tofacitinib in June 2024 due to inadequate control.

**Discussion:**

The difficulty in treating this patient have made us consider alternative diagnosis. MRI scans of her hips done in April 2023 showed bilateral coxa vara. This, along with no evidence of erosions and poor response to treatment, raised the possibility of Camptodactyly-arthropathy-coxa vara-pericarditis (CACP) syndrome. However, she did not have camptodactyly, her echocardiogram did not show pericardial effusion and the genetic testing was negative.

This patient has a complex social background. She lives with her mother, and no longer has contact with her father, or his side of the family. Her mother was involved in a car accident many years ago and due to a traumatic brain injury has difficulty with memory. Her mother has also suffered with depression. This, together with financial difficulties, have led to many missed appointments and concerns around compliance, despite continuous support from our specialist nursing team.

The patient has poor school attendance- more than can be accounted for by her disease. Her current school are very supportive. They give reminders about appointments, and a teacher collects her from home and takes her to school each day. When she starts secondary school this level of support will not be available.

Providing psychological support has been challenging due to her age and available resources. However, now she is 11 years old she will be able to get involved with our youth team. She struggles with needle phobia, and is very self-conscious about her swollen knees, and the sound they make when she moves.

At various times, safeguarding referrals have been made to try to support the family, none of which were accepted. Her mother’s reluctance to accept support from social services did not help either. This has presented the team with many challenges.

**Key learning points:**

• This is a challenging case of psoriatic JIA, unresponsive to multiple biologic agents, complicated by difficult social situation influencing care decisions. Non-attendance to clinics has contributed to delay in escalation of treatment at times. She was discussed at various regional and national MDT meetings, both in terms of diagnosis and management plans. Other differential diagnoses were considered and ruled out.

• The importance of addressing all the biopsychosocial aspects of care are highlighted in this case. The difficulty of balancing ongoing rapport and trust with the family, and ensuring safeguarding for the patient has been challenging.

**Learning points:**

• Importance of multi-agency and multi-centre working in management of patients with complicated JIA.

• Importance of difficult social situation affecting timely management decisions and the role of family support in optimising medical care.

• Even with newer treatments being approved on regular basis for JIA, it is important to recognise there are patients who prove resistant to many of these and more evidence is needed for combining biologics in such patients.

**OA23 Table 1.** DMARD and biologic treatments

Dates	Treatment Started	Treatment Stopped	Reason
September/2018	S/c methotrexate		Polyarthritis
January/2019	S/c adalimumab		Inadequate response to Methotrexate
March/2020		S/c Methotrextae S/c adalimumab	Concerns about compliance-change to IV medications
April/2020	IV methotrexateIV infliximab 4mg/kg		
February/2021	IV infliximab increased to 6mg/kg		Inadequate response
May/2021	IV infliximab increased to 8mg/kg		Inadequate response
December/2021		IV infliximab	Inadequate response to infliximab-escalate
January/2022	IV tocilizumab		
December/2022		IV methotrexate	Intolerance
March/2023		IV tocilizumab	Inadequate response to tocilizumab-escalate
April/2023	IV abatacept		
June/2023	Oral methotrexate		Added as adjunct to Abatacept
December/2023		IV abatacept Oral methotrexate	Inadequate response to Abatacept-escalate Intolerence to methotrexate
January/2024	Oral tofacitinib		
June/2024	S/c adalimumab		Inadequate response to tofacitinib alone

